# Newly Diagnosed Multiple Myeloma Patients with Skeletal-Related Events and Abnormal MRI Pattern Have Poor Survival Outcomes: A Prospective Study on 370 Patients

**DOI:** 10.3390/jcm11113088

**Published:** 2022-05-30

**Authors:** Nikolaos Kanellias, Ioannis Ntanasis-Stathopoulos, Maria Gavriatopoulou, Vassilis Koutoulidis, Despina Fotiou, Magdalini Migkou, Evangelos Eleutherakis-Papaiakovou, Panagiotis Malandrakis, Tina Bagratuni, Stylianos Mavropoulos-Papoudas, Maria Roussou, Efstathios Kastritis, Lia A. Moulopoulos, Meletios A. Dimopoulos, Evangelos Terpos

**Affiliations:** 1 Department of Clinical Therapeutics, School of Medicine, National and Kapodistrian University of Athens, 11528 Athens, Greece; nkanellias@med.uoa.gr (N.K.); johnntanasis@med.uoa.gr (I.N.-S.); mgavria@med.uoa.gr (M.G.); desfotiou@med.uoa.gr (D.F.); mmigkou@med.uoa.gr (M.M.); mdeleutherakis@gmail.com (E.E.-P.); panosmalan@med.uoa.gr (P.M.); tbagratuni@med.uoa.gr (T.B.); mroussou@med.uoa.gr (M.R.); ekastritis@med.uoa.gr (E.K.); mdimop@med.uoa.gr (M.A.D.); 2 1st Department of Radiology, School of Medicine, Aretaieion Hospital, National and Kapodistrian University of Athens, 11528 Athens, Greece; vkoutoulidis@med.uoa.gr (V.K.); lmoulop@med.uoa.gr (L.A.M.); 3 Health Data Specialists S.A., 11525 Athens, Greece; stymavropap@gmail.com

**Keywords:** multiple myeloma, skeletal-related events, MRI, bone, overall survival

## Abstract

Contemporary information is sparse on the frequency of skeletal-related events (SREs) in multiple myeloma (MM) patients at a population-based level in the era of novel agents. In this context, we conducted this single-center, prospective, observational study to determine the incidence of SREs among newly diagnosed MMs (NDMM) and to explore the possible correlations with disease characteristics, imaging finding, and patient prognosis. A total of 370 patients with available baseline MRIs were included. Among them, 208 (56%) presented with at least one SRE at diagnosis. Fractures were the most common reported SREs (48%). The incidence of SREs at diagnosis was higher in patients with osteolytic lesions, abnormal MRI pattern, hypercalcemia, and at least 60% bone marrow infiltration by plasma cells. Importantly, the patients with normal MRI pattern, who did not present with SREs at diagnosis, had statistically significant improved median OS in comparison with the patients who had abnormal MRI patterns and/or the presence of SREs at diagnosis (9.3 vs. 6.6 years, *p* = 0.048). Our data, which represent one of a few systematic reports on the incidence and characteristics of SREs in the era of novel agents, was indicative of a high incidence of SREs at the time of MM diagnosis. Early detection of myeloma bone disease and tailored patient management are essential to optimize patient outcomes.

## 1. Introduction

Multiple myeloma (MM) is the second most common hematologic malignancy, with approximately 86,000 newly diagnosed cases each year [1,2,3]. The median age at diagnosis is approximately 70 years [4,5]. Bone disease is one of the most common complications of MM, which also include anemia, hypercalcemia, infections, and renal failure [6]. Bone disease affects up to 80% of newly diagnosed MM patients (NDMM) [7], increasing the risk of development of skeletal-related events (SREs) [8]. SREs include pathologic fractures, requirement for radiotherapy or surgical intervention and spinal cord compression. 60% of MM patients will develop a fracture throughout the course of the disease [9]. The extent of bone disease correlates with disease burden, as well as with prognosis and quality of life (QoL) [10,11,12,13,14,15,16,17,18]. However, the impact of SREs on survival outcomes remains unclear.

The key role of bone disease in patient prognosis highlights the importance of imaging in the management of MM. Whole-Body Low-Dose CT (WBLDCT) was found to be superior in terms of detection of osteolytic lesions and has replaced Whole-Body X-rays (WBXR) in their detection [19,20,21,22,23,24,25]. According to the latest International Myeloma Working Group (IMWG) Guidelines, magnetic resonance imaging (MRI) is the gold standard imaging technique for the detection of bone marrow involvement in patients with symptomatic disease [26,27]. An MRI of the spine and pelvis can detect approximately 90% of the focal lesions in MM [26]. Evidence of bone marrow infiltration with MRI has a prognostic role as well; patients with diffuse patterns have poor outcomes [28,29,30].

International staging system (ISS) [31] and revised international staging system (R-ISS) [32] are the main predictive systems used for the survival assessment of patients with NDMM. Until today, the MRI pattern and presence of SREs at diagnosis were not incorporated in these staging systems. Additionally, although the frequency and characteristics of SREs in the MM patients who received conventional chemotherapy (CC) or thalidomide-based regimens along with bisphosphonates (BPs) were described, there are only scarce data available from the era of proteasome inhibitors (PI) or novel immunomodulatory agents (IMiDs) [33].

Thus, the aim of our study was to evaluate the incidence of SREs among the newly diagnosed patients with MM at diagnosis and at first relapse, along with the impact of SREs in survival outcomes. Additionally, we investigated the correlations among SREs, MRI pattern, patient, disease and treatment characteristics, and their impact on patient prognosis.

## 2. Materials and Methods

### 2.1. Study Design and Eligibility Criteria

This was a single center, prospective, observational study. The inclusion criteria included: (i) adult patients with NDMM; (ii) patients receiving first- and second-line therapy with novel agents (PI-, IMiD- or anti-CD38-based regimens); (iii) patients with available MRI examination at baseline; and (iv) patients who have given their written informed consent for recording the data from their medical records. The primary end point of the study was the evaluation of the incidence of SREs at diagnosis, and their impact on survival. Secondary end points included: (i) distribution of different types of SREs at diagnosis and during first or second relapse; (ii) possible correlations between the incidence of SREs with disease and patients’ characteristics.

### 2.2. Patient Enrolment

This study was conducted between February 2012 and February 2020 in a single center (Plasma Cell Dyscrasias Unit, Department of Clinical Therapeutics, National and Kapodistrian University of Athens, School of Medicine, Athens, Greece). Patients were informed about the objectives of the study before signing the informed consent and giving their approval for participation in the study. All study procedures were carried out in accordance with the declaration of Helsinki and its future amendments. All the procedures of the study were aligned with the regulations and guidelines pertaining to studies in Greece, as well as the Good Clinical Practice Guidelines (GCP), as defined by the international Council for Harmonization. The study was approved by the local Ethics Committee. Data were collected from prospectively maintained patient medical files. Treatment outcomes were recorded according to the IMWG criteria. 

### 2.3. Bone Disease: Imaging Studies and Assessment of SREs

Patients had a whole-body skeletal survey, using either conventional radiography (WBXR) or whole-body low-dose CT (WBLDCT) at diagnosis and then at the time of relapse or whenever clinically indicated. Patients with one or more osteolytic lesions in either WBXR or WBLDCT or at least one focal lesion in MRI were classified as patients with MM bone disease (MMBD) [27,34,35].

All of the patients had an MRI of the spine/pelvis at diagnosis. The MRI techniques used in our protocol included conventional and diffusion-weighted imaging (DWI-MRI) protocols. DWI-MRI was implemented for all of the patients from 1 January 2019 onward. Four MRI patterns were described. In a normal MRI pattern, no signal of abnormal intensity was present. In a focal MRI pattern, local areas of abnormal marrow intake were observed. A diffuse MRI pattern was characterized by the complete absence of normal bone marrow signal intensity. A variegated pattern was consistent with presence of several focal lesions on a background of intact marrow. A normal MRI pattern was associated with a low tumor burden. In cases of high tumor burden, the MRI had characteristic diffuse hypointense findings on T1 and diffuse hyperintensity on T2 images, with an additional enhancement after gadolinium administration [28].

Definition of SREs: Pathologic fractures were identified either during clinical evaluation of the patient due to localized pain, or incidentally. Bone radiotherapy was defined as radiation therapy for emergency spinal cord compression (SCC), or as palliative therapy due to pain. Surgical procedures included all of the therapeutic interventions required for correction of spinal cord compression or pathologic fractures. Main surgical interventions included vertebroplasty, balloon kyphoplasty, and laminectomy. Those minimally invasive procedures have shown to be effective regarding pain relief and restoration of function in the patients who do not respond to conservative measures [36]. SCC was identified using either CT or MRI of the respective spinal region, according to institutional Guidelines. SREs identified within 30 days of MM diagnosis were classified as baseline SREs. Patients who presented with a SRE of a single category, were considered as patients with a single SRE, while the patients who presented with SREs of two or more categories, were considered as patients with combinations of SREs.

### 2.4. Statistical Analysis

For the analysis of this data, the R version 4.0.4 (Bell Laboratories, Norcross, GA, United States) (15 February 2021) was used. The IDE selected was RStudio version 1.4.1103. During the study, several contingency tables were assessed for significance. The tests used were the chi-square test and Fisher’s exact test. The tests were implemented using R functions “chisq.test” and “fisher.Test” from the R package stats version 3.6.2. Regarding survival analysis, the R survival package v2.11-4 was used. The Kalbfleisch–Prentice estimate was used to fit the survival curves. This method becomes equivalent to the Kaplan–Meier estimation when the weight functions reduce to unity. To compare the survival curves, the G-rho family of Harrington and Fleming was implemented in R by the survdiff function, specifically the log-rank or Mantel–Haenszel test. Plots were produced with the survminer package v0.4.8. The survival analysis was performed for two groups of the patients from the original dataset. The presence or not of bone disease was the discriminating factor. The absence of bone disease (bone condition is normal) was determined by a lack of SREs in combination with a normal MRI pattern. The presence of bone disease (bone condition is abnormal) was determined by an abnormal MRI pattern, or the presence of SREs at diagnosis. Abnormal MRI pattern was defined as the presence of focal, diffuse, or salt and pepper bone marrow penetration. An additional grouping, where abnormal MRI pattern was defined as focal and diffuse without including the variegated tissue penetration, was also analyzed.

## 3. Results

### 3.1. Patient Characteristics

Patients’ baseline characteristics are shown in Table 1. Overall, data from the 370 patients with NDMM according to the IMWG criteria are included in the present analysis. A total of 200 (54%) of them were males, and 99% were Caucasian. The median age at diagnosis was 65 (range 31–92). One third were ISS stage 1 (34%), one third were ISS stage 2 (35%), and another third were ISS stage 3 (31%). A total of 214 patients (58%) had IgG myeloma subtype, 90 patients had IgA (24%), and 62 patients had light-chain myeloma (17%). The majority of patients (*n* = 220, 60%) had ECOG performance status (PS) 0 or 1 at diagnosis. One hundred and twenty patients (*n* = 120, 32%) were classified as capable of only limited self-care or were completely disabled (ECOG PS: 3–4). Interestingly, this was mainly attributed to myeloma bone disease complications (105/120 patients). Only 15/120 patients had limitations in the activities of daily living due to other conditions (stroke, dementia, chronic inflammatory diseases). All of the patients received bone-targeted agents, except for those with creatine clearance less than 30 mL/min. Overall, 311 patients received zoledronic acid, whereas only 19 patients received denosumab. Regarding the type of first line treatment, 152 patients received PI-based regimens. A total of 163 patients received IMID-based treatment. A total of 26 patients received PI- and IMID-based combinations, whereas 29 received conventional chemotherapy.

### 3.2. Myeloma Bone Disease at Diagnosis

At diagnosis, WBXR was performed in 344/370 of the patients and WBLDCT in 95/370 patients; 71 of the patients had both WBXR and WBLDCT (Table 2). Overall, 294 (80%) of the patients presented with at least one lytic lesion in either WBXR or WBLDCT at diagnosis. Evidence of osteolytic disease, defined as more than one osteolytic lesion, was present in 271/344 (79%) of the patients according to WBXR, and in 83/95 (87%) of the patients by means of WBLDCT. MRI results of the spine and pelvis at diagnosis were available in all 370 of the patients; 151 (40%) of the patients had a focal, 139 (38%) of the patients had diffuse, 58 (16%) of the patients had normal, and 22 (6%) of the patients had variegated patterns of marrow involvement.

We also evaluated the presence of osteopenia and osteoporosis in the DXA scan of the femoral neck and spine in 59 patients who had signs of osteoporosis in the WBXR/WBLDCT at the time of MM diagnosis. Among this subgroup of patients, 13 patients had a normal DXA scan, 27 had osteopenia, and 19 had osteoporosis. All 46 of the patients with abnormal findings in the DXA scan had one or more osteolytic lesions on WBXR or WBLDCT.

### 3.3. SREs’ Incidence at Diagnosis and at Relapse

Overall, 208 (56%) of the patients presented with one or more SREs at diagnosis. A total of 176 NDMM patients (48%) presented with one or more pathological fractures, with a total of 254 documented fractures. A total of 23 (6%) received radiotherapy, 27 (7%) underwent surgery, and 29 (8%) experienced SCC. More specifically, 168 out of the 370 patients (45%) presented with a single SRE (Table 3). Among them, 146 (87%) presented with one or more pathological fractures, while 7 (4%) needed radiotherapy, mainly for palliative reasons, 7 (4%) of the patients underwent bone surgery, whereas 8 (5%) of the patients presented with SCC due to external pressure by bone plasmacytomas. Furthermore, 40 of the patients (11%) presented with combinations of SREs subtypes (Table 3). The most frequent combinations were fracture with surgery or SCC. Surgical management included mainly kyphoplasty and laminectomy. A thorough description of SREs loci at diagnosis is presented in Table 4. The most common sites of fractures at MM diagnosis were the lower thoracic spine, the upper lumbar spine and the ribs. The distribution of SREs according to baseline disease characteristics is provided in Table 5. Neither ISS nor R-ISS stage were associated with the incidence of SREs at baseline.

In our study, 240 of the patients received second line therapy after documented disease progression. Among them, 57 (24%) of the patients presented with SREs at relapse. A total of 36 of the patients had pathological fractures, 19 required radiotherapy, 1 bone surgery, and 1 patient showed signs of SCC. Data were also available for 154 of the patients with relapsed/refractory MM (RRMM) who received a third line of treatment. Among them, 29 of the patients (19%) presented with SREs at second relapse, 12 with pathological fractures, 11 required radiotherapy, 2 required surgery to bone, and 4 of the patients presented with signs and symptoms of SCC.

### 3.4. SREs/MRI Pattern Correlations with Patient and Disease Characteristics

The incidence of SREs at diagnosis was higher in the patients with osteolytic lesions either on WBXR (*p* < 0.001) or WBCT (*p* = 0.013) than those without lytic lesions, abnormal MRI pattern (*p* < 0.001) than those with normal pattern, those with hypercalcemia (*p* = 0.023), and those with at least 60% bone marrow infiltration by plasma cells (*p* = 0.032). No statistically significant associations emerged between the presence of single or multiple SREs at diagnosis and baseline characteristics. Regarding the MRI findings, NDMM patients with an abnormal MRI pattern were more likely to present with more advanced disease, according to ISS (*p* < 0.001) and R-ISS (*p* < 0.001), hypercalcemia (*p* < 0.01), anemia (*p* < 0.001), and bone marrow infiltration by plasma cells of 60% or greater (*p* < 0.001).

### 3.5. Prognostic Factors for Survival

The presence of SREs at diagnosis was not associated with inferior overall survival (OS) (*p* = 0.19). Furthermore, we did not find any statistically significant correlation between the presence of a single SRE versus multiple SREs at diagnosis and OS (*p* = 0.77).

Regarding MRI findings at diagnosis, no statistically significant difference emerged between the patients with focal or diffuse MRI pattern and those with normal MRI pattern (*p* = 0.14 and *p* = 0.13, respectively).

**Figure 1:**
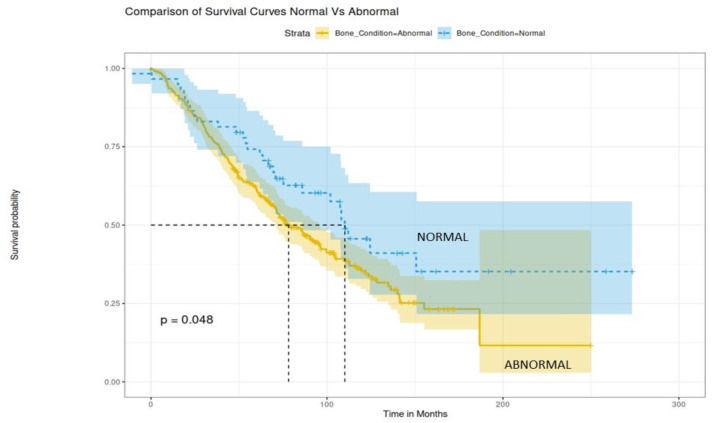
OS curves according to bone condition at MM diagnosis. Normal bone condition signifies absence of SREs and normal MRI pattern, whereas abnormal bone condition signifies presence of at least one SRE and/or abnormal MRI pattern.

We then compared survival outcomes among the patients with normal bone condition at diagnosis (absence of SREs and normal MRI pattern) and patients with abnormal bone condition (SREs and/or abnormal MRI pattern). Interestingly, the patients with a normal bone condition had a 74% 5-year OS rate with a median OS of 112 months (9.3 years), whereas patients with abnormal bone condition had a 62% 5-year OS rate and a median OS of 80 months (6.6 years) (*p* = 0.048, Figure 1). We also performed a sensitivity analysis by excluding patients with a variegated MRI pattern and we found similar results (*p* = 0.046).

## 4. Discussion

Bone disease is one of the most important features of MM. In our study, 294/370 (79%) of the patients presented with at least one lytic lesion, either in WBXR or WBCT, and 312/370 (84%) presented with an abnormal MRI pattern at diagnosis. Our results are in line with the existing literature in the field [12].

SREs represent the main manifestation and one of the hallmarks of MMBD. However, novel anti-myeloma agents exert a favorable effect on bone metabolism [37,38]. In this study we evaluated data from a single center to describe the incidence of SREs in the real-world setting, in the era of novel agents. Our data, which represent one of a few systematic reports on the incidence and characteristics of SREs in the era of novel agents, was indicative of a high SREs incidence at MM diagnosis. Overall, 208/370 (56%) of the patients with NDMM presented with one or more SREs at diagnosis. This highlights the crucial role of SREs as a major factor in the burden of bone disease in NDMM patients. The leading cause of SREs were fractures, which necessitate a multidisciplinary management that may include surgery, radiotherapy, and/or palliative care.

A few studies have also examined the incidence of SREs at MM diagnosis. Our results are in concordance with these data. In a retrospective cohort study conducted in the United States among the 343 patients with NDMM, 41.5% experienced at least one SRE at diagnosis [33]. In another population-based study including 1112 patients with NDMM, a 32% incidence of SREs was reported [39].

Furthermore, in our analysis, we aimed to explore and identify several disease and patient characteristics, which may correlate with SREs. In this context, we found that the incidence of SREs at diagnosis was higher in the patients with osteolytic lesions, abnormal MRI pattern, and elevated calcium levels. The above-described characteristics are indicative of a high burden of bone disease. Our data highlighted the impact of bone disease burden as an essential trigger in the development of SREs [9].

Fractures are the most frequently studied of the SREs in the patients with MM. In a large study, which included 66,079 patients with MM, the presence of fracture was related to inferior survival outcomes [40]. In another case control study, 49 of the patients with NDMM were evaluated in terms of survival, based on the presence of fractures at diagnosis. The presence of fractures had a negative impact on survival [41]. The negative impact of SREs on patient outcomes was also described for patients with prostate and breast cancer [42,43].

The impact of MRI pattern at diagnosis was thoroughly investigated in the current literature in terms of survival. In the era of conventional chemotherapy, a study evaluated 142 of the patients with NDMM. Patients with diffuse MRI pattern had inferior median OS, in comparison with the patients with normal, focal, or variegated pattern [28]. In another study, which included the patients receiving conventional chemotherapy or thalidomide, the diffuse and variegated patterns were identified as independent factors of disease progression [44]. In the era of novel agents, diffuse MRI pattern has also shown a negative impact on survival. Additionally, a subgroup of patients with diffuse MRI pattern, advanced disease stage (ISS 3), and high-risk cytogenetics demonstrated very poor survival outcomes (median OS of 21 months and 3 year OS probability of 35%) [29]. Interestingly, the patients with at least three large focal lesions have inferior survival outcomes, independent of the R-ISS stage [45]. Furthermore, focal lesions identified by PET/CT at baseline have a strong prognostic value in combination with the R-ISS stage in the patients with NDMM [46]. Our analysis indicated that the co-existence of a normal MRI pattern and absence of SREs at diagnosis led to a superior OS, compared with abnormal MRI pattern and/or the presence of SREs at diagnosis. It has to be mentioned that an abnormal MRI pattern itself was not associated with a statistically significant inferior OS compared with the normal MRI pattern at baseline. This may be attributed to confounding factors, including the differences in the upfront treatment regimens used in our study compared with previously published data encompassing more patients who received PI- and IMiD-based combinations in the first line.

Our study has several limitations. The first one is the method of bone disease detection. Most of our patients had WBXR, while a limited number of the patients had WBLDCT at diagnosis. In the rapidly evolving field of imaging in MMBD, even more accurate methods are available for the detection of osteolytic lesions, such as PET-scan or WBMRI. Furthermore, the fact that all of the data came from a single tertiary center may also introduce a referral or selection bias. For example, all of the patients had similar ethnic and race characteristics. Broader cohorts are needed to establish the role of SREs and their impact on bone disease pathophysiology and patient outcomes. According to the current treatment guidelines for the patients with NDMM, triplet or quadruplet regimens are generally recommended as frontline therapy [47]. However, less than 10% of the patients had received such combinations in the upfront setting in our study, due to differences in the standard of care in the real-world practice in our country during the time period of the study. In addition to the above, the small number of the patients who received denosumab did not allow for a subgroup analysis on the impact of bone targeting agents (bisphosphonates, denosumab) on survival, according to the bone condition at baseline. We also did not include data on extramedullary disease because not all of the patients performed WBCT or PET/CT scan in order to have a robust assessment of EM disease at baseline. The above limitations may prevent the generalizability of our results. Data from prospective or retrospective studies are needed in order to incorporate SREs and/or abnormal imaging findings in prognostic models for the patients with MM.

In conclusion, our study indicated that more than 50% of our patients presented with SREs at diagnosis. The presence of SREs and/or abnormal MRI pattern was associated with inferior survival. Early detection and prompt management of SREs are essential. Currently, the main staging systems for MM are ISS and R-ISS stage. However, these two widely used systems have not incorporated MM bone disease assessment or SREs. Our results indicate that, in addition to the evaluation of standard clinical risk factors included in the ISS and R-ISS, we have to identify and closely monitor other markers of disease burden of potential prognostic value. Current guidelines recommend a baseline imaging assessment in every patient with NDMM including novel techniques, such as WBCT, PET/CT scan and WBMRI. Imaging results should be thoroughly and carefully interpreted by experts, in order to identify patient groups with SREs, or those at high risk for developing SREs, who may necessitate a tailored treatment and management plan.

## Figures and Tables

**Table 1:** Baseline patient characteristics (*n* = 370).

➢ **SEX:** 170F (46%) 200M (54%)
➢ **RACE/ETHNICITY**
○ Caucasian: 366 (99%)
○ Black/African American: 4 (1%)
➢ **AGE AT DIAGNOSIS**
○ 20–40: 14 (4%)
○ 41–60: 126 (34%)
○ 60–80: 206 (56%)
○ >81: 24 (6%)
➢ **ECOG PS**
○ 0: 120 (33%)
○ 1: 100 (27%)
○ 2: 30 (8%)
○ 3: 70 (19%)
○ 4: 50 (13%)
➢ **ISS STAGE AT DIAGNOSIS**
○ 1: 124 (34%)
○ 2: 128 (35%)
○ 3: 118 (31%)
➢ **R-ISS STAGE AT DIAGNOSIS (*n* = 340)**
○ 1: 87 (26%)
○ 2: 171 (50%)
○ 3: 82 (24%)
➢ **MULTIPLE MYELOMA SUBTYPE**
○ IgG: 214 (58%)
○ IgA: 90 (24%)
○ IgM: 2 (0.5%)
○ IgD: 2 (0.5%)
○ κ-light chain: 34 (9%)
○ λ-light chain: 28 (8%)
➢ **CYTOGENETICS**
○ Del 17p: 31 (8%)
○ t (4; 14): 31 (8%)
○ t (14; 16): 8 (2%)
○ t (11; 14): 17 (5%)
○ del13q: 106 (29%)
○ add 1q21: 75 (20%)
➢ **BONE MARROW INFILTRATION**
○ PLASMA CELLS 10–30%: 67 (18%)
○ PLASMA CELLS 31–60%: 93 (25%)
○ PLASMA CELLS 61–100%: 210 (57%)
➢ **ANEMIA**
○ Hgb < 10 g/dL: 153 (41%)
○ Hgb 10–12 g/dL: 122 (33%)
○ Hgb > 12 g/dL: 95 (26%)
➢ **RENAL FUNCTION**
○ Cr < 1.2 mg/dL: 272 (74%)
○ Cr 1.3–3 mg/dL: 68 (18%)
○ Cr > 3 mg/dL: 30 (8%)
○ CrCl < 30 mL/min: 40 (11%)
○ Patients requiring dialysis: 25 (7%)
➢ **HYPERCALCEMIA** (Corrected Calcium > 11.5 mg/dL): 45 (12%)
➢ **FRONTLINE THERAPY**
○ PI-based: 152 (41%)
○ IMID-based: 163 (44%)
○ PI- and IMID-based: 26 (7%)
○ Conventional chemotherapy: 29 (8%)

**Table 2:** Description of bone involvement at MM diagnosis.

Osteolytic Lesions by WBXR (*n* = 344)	No lesions	1–3 lesions	More than 3 lesions	
	73 (21%)	48 (14%)	223 (65%)	
Osteolytic Lesions by WBLDCT (*n* = 95)	No lesions	1–3 lesions	More than 3 lesions	
	12 (12%)	7 (8%)	76 (80%)	
MRI PATTERN (*n* = 370)	NORMAL	FOCAL	DIFFUSE	VARIEGATED
	58 (16%)	151 (40%)	139 (38%)	22 (6%)
DXA SCAN (*n* = 59)	NORMAL	OSTEOPENIA	OSTEOPOROSIS	
	13 (22%)	27 (46%)	19 (32%)	

**Table 3:** Incidence of SREs at MM diagnosis.

SINGLE SKELETAL-RELATED EVENTS (*n* = 168)	
RADIOTHERAPY	7 (4%)
FRACTURE	146 (87%)
SURGERY	7 (4%)
SPINAL CORD COMPRESSION	8 (5%)
SRE COMBINATIONS (*n* = 40)	
RADIOTHERAPY—FRACTURE	5 (12.5%)
FRACTURE—SURGERY	11 (27.5%)
RADIOTHERAPY—SPINAL CORD COMPRESSION	3 (7.5%)
RADIOTHERAPY—SURGERY	1 (2.5%)
SURGERY—SPINAL CORD COMPRESSION	1 (2.5%)
FRACTURE—SPINAL CORD COMPRESSION	12 (30%)
FRACTURE—SURGERY—RADIOTHERAPY	2 (5%)
SPINAL CORD COMPRESSION—SURGERY—RADIOTHERAPY	5 (12.5%)

**Table 4:** Distribution of SREs at diagnosis.

	FRACTURE	RADIOTHERAPY	SURGERY	SPINAL CORD COMPRESSION
CERVICAL SPINE				
C1				
C2		1		
C3	2	3		2
C4				
C5	2		1	1
C6			2	2
C7			1	
THORACIC SPINE				
T1				
T2	1			3
T3	3			2
T4	6		1	1
T5	4			1
T6	14	1	1	7
T7	8	1	2	2
T8	17		2	1
T9	8			2
T10	8	1		
T11	23	2	2	3
T12	28	1	1	1
LUMBAR SPINE				
L1	21		1	
L2	22	1	3	1
L3	14	1	1	
L4	17	2	3	
L5	5	3	2	
RIBS	33			
STERNUM	2			
PELVIS	5	3	2	
SACRUM	1			
CLAVICLE	7			
HUMERI	3	1	2	
FEMUR			1	
TIBIA	1			

**Table 5:** Distribution of SREs according to baseline disease characteristics.

	SRES YES	SRES NO
OSTEOLYTIC LESIONS WBXR		
YES (*n* = 271)	147	124
NO (*n* = 73)	21	52
OSTEOLYTIC LESIONS WBLDCT		
YES (*n* = 83)	57	26
NO (*n* = 12)	2	10
MRI PATTERN		
NORMAL (*n* = 58)	0	58
ABNORMAL (*n* = 312)	183	129
BONE MARROW INFILTRATION		
10–60% (*n* = 160)	79	81
>60% (*n* = 210)	102	108
HYPERCALCEMIA		
NORMAL CALCIUM (Corrected Calcium < 11.5 mg/dL) (*n* = 325)	150	175
ELEVATED CALCIUM (Corrected Calcium >11.5 mg/dL) (*n* = 45)	39	6
ISS STAGE		
ISS I (*n* = 124)	57	67
ISS II (*n* = 128)	63	65
ISS III (*n* = 118)	63	55
R-ISS STAGE		
R-ISS I (*n* = 87)	37	50
R-ISS II (*n* = 171)	83	88
R-ISS III (*n* = 82)	48	34

## Data Availability

Raw data are available from the authors upon reasonable request.
